# Metal oxide heterojunctions using a printable nickel oxide ink†

**DOI:** 10.1039/c9ra08466e

**Published:** 2020-01-23

**Authors:** Hari Ramachandran, Mohammad Mahaboob Jahanara, Nitheesh M. Nair, P. Swaminathan

**Affiliations:** Electronic Materials and Thin Films Lab, Department of Metallurgical and Materials Engineering, Indian Institute of Technology Madras Chennai 600036 India swamnthn@iitm.ac.in; Organic Electronics Group, Department of Electrical Engineering, Indian Institute of Technology Madras Chennai 600036 India

## Abstract

Wide band gap metal oxides are ideally suited for inorganic optoelectronic devices. While zinc oxide is a commonly used n-type material, there is still a lot of ongoing work for finding suitable p-type oxides. In this work, we describe a two-step route to formulate a stable and conducting p-type nickel oxide (NiO) nanofluid. NiO nanoparticles were synthesised using a bottom-up wet chemical approach and dispersed in ethylene glycol to form a nanofluid. The viscosity and surface tension of the nanofluid were optimised for printing. The printing was done using an extrusion-based direct writer. The NiO nanofluid was printed onto an aluminum-doped zinc oxide layer and annealed at different temperatures. Electrical characterisation of the junction was used to extract the junction barrier for carriers across the interface. The resulting heterojunction was found to exhibit rectifying behaviour, with the highest rectification ratio occurring at an annealing temperature of 250 °C. This annealing temperature also resulted in the lowest junction barrier height, and was in excellent agreement with theoretically predicted values. The development of a printed p-type ink will help in the realisation of oxide-based printed electronic devices.

## Abstract 2

We present a printed p-NiO/n-AZO heterojunction exhibiting high rectification ratios using a NiO ink synthesized by a chemical route.

## Graphical abstract

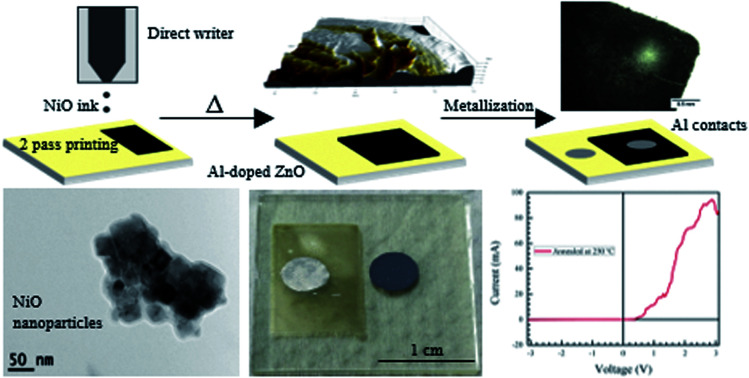

## 1 Introduction

Advances in the development of transparent n-type materials such as zinc oxide (ZnO), tin oxide (SnO_2_), and indium oxide (In_2_O_3_) have led to strides being taken towards the realisation of all-oxide large scale microelectronic devices through wet chemical techniques.^1^ However, a lack of availability of high-performance p-type materials has served as a constraint for certain applications, leading to the use of either unipolar devices or choosing a combination of inorganic–organic hybrid devices.^2,3^ The realisation of p-type materials with properties such as hole mobilities and transparencies similar to their n-type counterparts, and that are easy to process would lead to the development of transparent devices and displays, with the potential to impact several facets of daily life.^3^

The main difficulty in achieving similar performance in p-type materials arises from the difference in mechanism of the formation and conduction of holes. In n-type metal oxides, oxygen vacancies produce electrons for conduction.^4^ However, in p-type oxides, hole creation is limited by the formation energy of point defects that act as hole producers (such as oxygen interstitials and metal vacancies), the ionization energy of these defects to release holes, and the formation energy of native defects that act as hole killers (such as cation interstitials and anion vacancies).^5^ These effects pose challenges in the realisation of stable p-type oxides that are convenient to process.^6^

In recent years, inkjet printing has emerged as a cost-effective technique for fabricating electronic devices with high quality, reliability, and minimal wastage. The move from continuously-dispensing systems, where ink is diverted to a reservoir when not required, to drop-on-demand (DOD) systems facilitates lower costs as the ink is ejected only when required. DOD systems also help in conserving costly precursor material.^7^ Some of the other advantages of printing as a route for device fabrication include the short deposition time, compactness, lack of a patterning step, and adaptability to various substrates.^8^

Inkjet printing requires the synthesis of stable and jettable inks. Inks typically consist of an active agent, such as graphene, carbon nanotubes, metal nanoparticles, or polymers, and a dispersing medium.^9^ Surfactants can be also be added to increase the stability of inks by preventing coalescence of the individual nanostructures. But, they tend to reduce the conductivity of the printed patterns as the surfactants hinder connectivity between the particles and hence they need to be removed post-printing.^8^

Nickel oxide (NiO) is a transition metal oxide with rock salt structure. It has a wide bandgap ranging from 3.6–4 eV and a work function of around 5.2 eV.^10,11^ Stoichiometrically pure NiO is green in colour, while the presence of nickel vacancies (V_Ni_) causes it to be black, and also imparts a stable p-type character to the material.^12,13^ NiO thin films have been deposited by a variety of physical routes (such as magnetron sputtering,^14–16^ pulsed laser deposition,^17^ and electron beam deposition^18^) and chemical routes (including sol–gel processing,^19^ spray pyrolysis,^20^ and atomic layer deposition^21^). NiO shows promise in myriad applications, such as thin film transistors,^22–25^ pn junctions,^26–29^ electrochromic displays,^30^ resistive switching memory,^31^ optoelectronic,^32,33^ and photovoltaic^17,33–35^ devices. NiO based printed devices have been used in several applications, such as pesticide detection,^36^ as thermistors,^37^ and as hole-transport layers in solar cells.^38^

Pure ZnO is a wide bandgap semiconductor, with a direct gap of 3.37 eV, and a work function of 4.65 eV.^8,39^ The presence of oxygen vacancies (V_O_) causes shallow donor states near the bottom of the conduction band. This renders ZnO as an intrinsically n-type semiconductor. Aluminum-doped zinc oxide (AZO) has better conductivity, stability, and transparency when compared to undoped ZnO.^7,8,40^ It is a degenerate semiconductor, with its Fermi level being close to the conduction band minimum, making its work function equal to its electron affinity (4.65 eV).^41^ AZO also finds application as a transparent conducting oxide, with the potential to replace expensive indium tin oxide.

In this work, we describe the synthesis of phase pure, nanosized NiO using a bottom-up chemical approach, and the subsequent formulation of a surfactant-free NiO–ethylene glycol (EG) based printable ink. The ink was printed using a custom built direct writer onto AZO coated glass substrates, and the heterojunction annealing conditions were optimised. The phase purity of the nanoparticles was obtained using X-ray diffraction (XRD) and using Fourier-transform infrared spectroscopy (FTIR). The particles were imaged using transmission electron microscopy (TEM) and scanning electron microscopy (SEM). The Raman spectrum of the synthesised NiO was studied to characterise the defects in the material. The absorption spectrum of NiO was measured to obtain the optical gap of the material. The viscosity, surface tension, and contact angle of the synthesised ink were measured to ascertain its printability and wettability. The *I*–*V* response of the printed heterojunction was studied using a four-probe setup, and the junction's morphology was imaged using optical microscopy and profilometry. We found that rectification ratios in excess of 1000 could be achieved using suitable printing (60 °C) and junction annealing temperatures (250 °C). The values of the junction barrier height were found to be in excellent agreement with theoretically predicted values, for junctions annealed at this temperature. This work provides a step towards the development of printable metal oxide heterojunctions.

## 2 Experimental procedure

### 2.1 Materials

All chemicals were of reagent grade and used without any further purification. Nickel chloride hexahydrate (NiCl_2_·6H_2_O), hydrazine monohydrate (N_2_H_4_·H_2_O), potassium hydroxide (KOH), acetone, and ethanol were obtained from Sigma-Aldrich. The AZO coated glass slides were purchased from Techinstro Industries. The AZO layer had a thickness of 800–850 nm, sheet resistances of 8–10 Ω sq^−1^, and transparency of approximately 80% in the 400–1000 nm range.

### 2.2 Synthesis of NiO nanoparticles

NiO nanoparticles were synthesised by the thermal decomposition of nickel hydroxide (Ni(OH)_2_) through a modification of the technique reported by El-Kemary *et al.*^42^ The method relies upon the complex forming ability of the Ni^2+^ ion. Nickel forms a complex with hydrazine in an ethanolic medium, and on the action of a strong base, precipitates out as green nickel hydroxide. The hydroxide is dried before being annealed to convert it to NiO.

A 0.11 M solution of nickel chloride hexahydrate was prepared in absolute ethanol. Hydrazine monohydrate was added such that [Ni^2+^/N_2_H_4_] = 5. The pH of the mixture was adjusted to 12 using KOH (1 M solution). The mixture was stirred for 2 h at room temperature and was allowed to rest for a further 2 h. The solid component of the mixture was collected using a centrifuge, and was washed multiple times with a 0.1 M solution of KOH in deionized water and then with acetone. The resulting bright green material was dried in an oven at 80 °C for 5 h and annealed in a furnace at 400 °C for 2 h. The black material obtained was then ground to a fine powder using a mortar and pestle.

### 2.3 Formulation of NiO nanoink

Based on a previous work, a 4 vol% dispersion of metal oxide nanoparticles in EG was found to have optimal properties for inkjet printing.^8^ The same formulation was used here. NiO powder was carefully weighed and then added to EG to obtain the required vol%. The solution was sonicated for 3 h using a Branson 2800 bath ultrasonicator to ensure uniform dispersion of the NiO particles in EG.

Glass slides were cleaned by sonicating in ethanol, acetone, isopropyl alcohol, and deionized water. The ink was dropcast on the slides using a micropipette. The dropcasting was done at a range of temperatures (30–60 °C) to observe how it affected the uniformity of the pattern. A dwell time of 10 min was allowed, after which the slides were baked at 120 °C for one hour to remove the solvent from the dropcast patterns.

### 2.4 Direct writing of the nanoink

The printing of the ink was done using a custom-built direct writer, manufactured by Tvasta Manufacturing Solutions, Pvt. Ltd. The ink is loaded into a commercially available medical syringe, from where it is extruded out of a needle (0.2 mm diameter). The syringe can move in the *x*-direction while the substrate bed can move in the *y*-direction. A plunger, moving in the *z*-direction, controls the extrusion of the ink. The substrate bed can also be heated up to 70 °C during deposition. The movement of the syringe and bed is controlled by Repetier-G software. Further details about the printer and the printing process are available in the ESI.† The direct writer setup is shown in Fig. S1.†

A 1.5 × 1.5 cm filled square pattern was loaded into the software and the ink was printed onto cleaned glass slides. The patterns were then dried to remove the excess solvent, and annealed to facilitate connectivity of the nanoparticles in the printed layer. Table 1 gives the parameters used in the printing. The optimisation of these parameters has been described elsewhere.^8^

**Table 1:** Parameters used for printing onto glass and AZO coated glass

Parameter	Glass substrate	AZO glass substrate
Printing volume	210 μL	280 μL
Number of printed layers	1	2
Bed temperature	30 and 60 °C	60 °C
Drying temperature	120 °C	120 °C
Drying time	1 h	1 h
Annealing temperature	250 °C	200, 250 & 300 °C
Annealing time	2 h	2 h

### 2.5 Formation of the heterojunctions

AZO coated glass slides were cleaned by sonicating in ethanol, isopropyl alcohol, and deionized water. A 1.5 × 1 cm filled pattern was loaded into the software. After printing and drying, the patterns were annealed at different temperatures to observe its effect on the *I*–*V* characteristics of the heterojunction. The printing parameters are shown in Table 1. Aluminum contacts (350 nm thickness) were then deposited on both NiO and AZO using thermal evaporation. A suitable mask was used to isolate the contacts. Aluminum contacts were also deposited onto a plain AZO coated slide in order to verify that the aluminum–AZO junction was ohmic in nature.

### 2.6 Characterisation equipment

Crystallographic information was obtained by XRD, Xpert Pro PANalytical diffractometer, with Cu-Kα radiation of wavelength 0.154 nm. The morphology of the as-synthesised particles was analyzed by TEM, Tecnai F20, FEI, operating at an accelerating voltage of 200 kV. The agglomeration of the nanoparticles was imaged using SEM, Quanta 400, operating at an accelerating voltage of 20 kV. The infrared absorption was obtained using a Jasco FT/IR-6300 Infrared Spectrometer. The Raman spectrum was obtained using a Witec UHTS 300 Spectrometer, with an excitation wavelength of 514.5 nm. The absorption spectrum was obtained in the 200–800 nm range, using a Jasco 650 Spectrometer. The viscosity of the nanofluid was measured using a rheometer, Physical MCR 301, Anton Paar, over a shear rate range of 100–1000 s^−1^ at room temperature. Parallel plate geometry was used for this, with a plate diameter of 25 mm. The surface tension of the ink and the contact angle of the ink deposited on glass and AZO coated glass was obtained using an Apex Acam-D3 Contact Angle Meter. The height of the printed patterns and surface roughness were measured using an AEP Tech Nanomap 1000 WLI optical profiler. Optical micrographs of the dropcast and printed patterns were obtained using a Metallic 3 Leitz Wetzlar Microscope. Current–voltage measurements were made using a Keithley 4200A-SCS parameter analyzer.

## 3 Results and discussion

### 3.1 Characterisation of NiO nanoparticles

**Fig. 1:**
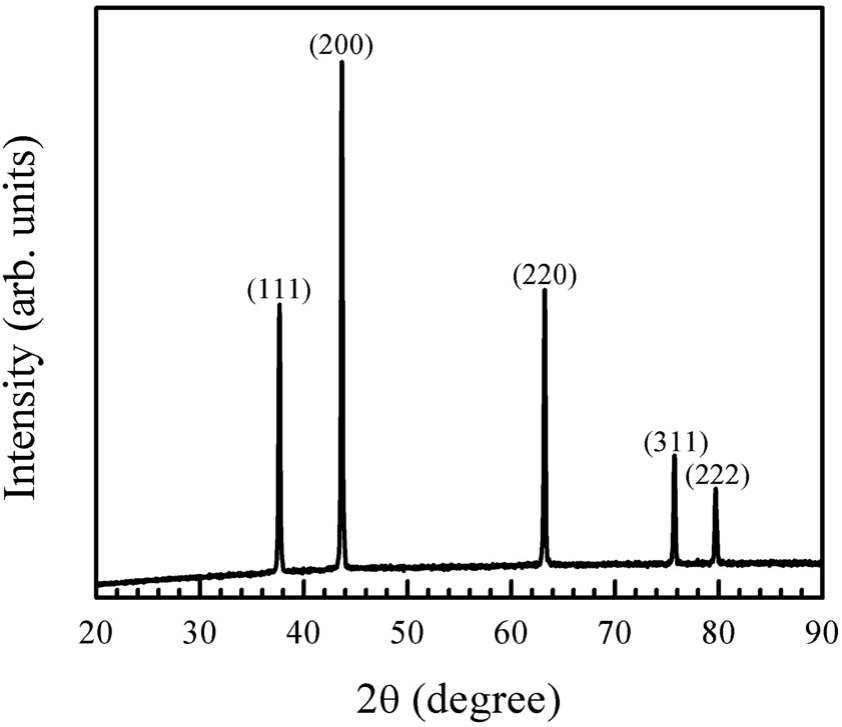
X-ray diffraction pattern of the synthesised nanocrystalline particles matches the ICDD file for NiO (ICDD card number 98-005-2854).

The chemical synthesis route adapted here involves the preparation of nickel hydroxide, which is then converted into NiO by annealing at low temperature (400 °C for 2 h). The XRD pattern of the annealed powder is shown in Fig. 1. The peaks in the diffraction pattern correspond to crystalline cubic NiO (ICDD card number 98-005-2854). Scherrer formula was used to obtain the crystallite size, using the (200) peak, which had the highest intensity. The crystallize size was found to be 50 nm.

**Fig. 2:**
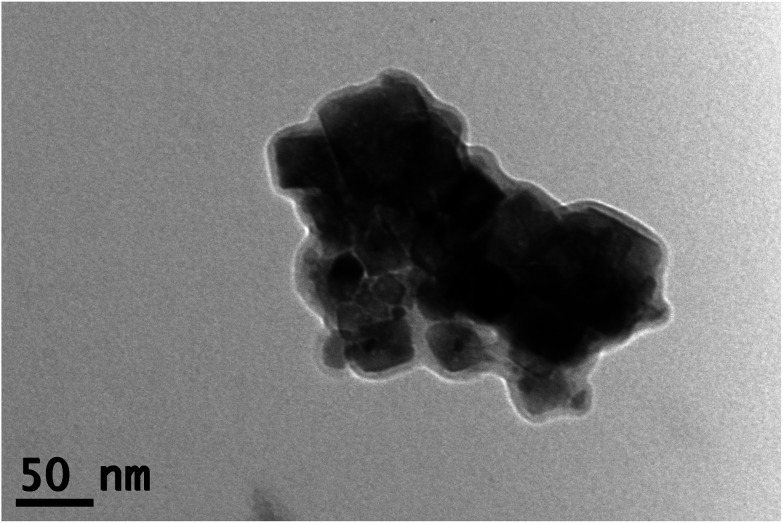
TEM image of the NiO nanoparticles. Individual particle sizes ranged from 15 to 65 nm, corresponding to the size obtained from Scherrer formula, which was approximately 50 nm.

Fig. 2 shows a representative TEM image of the NiO nanoparticles. The spread of individual particle sizes was found to be approximately 15–65 nm, which was in agreement with the crystallite size obtained from Scherrer formula. The particles were observed to have a tendency to agglomerate (Fig. S2 in ESI†), since no surfactant was added during the synthesis.

**Fig. 3:**
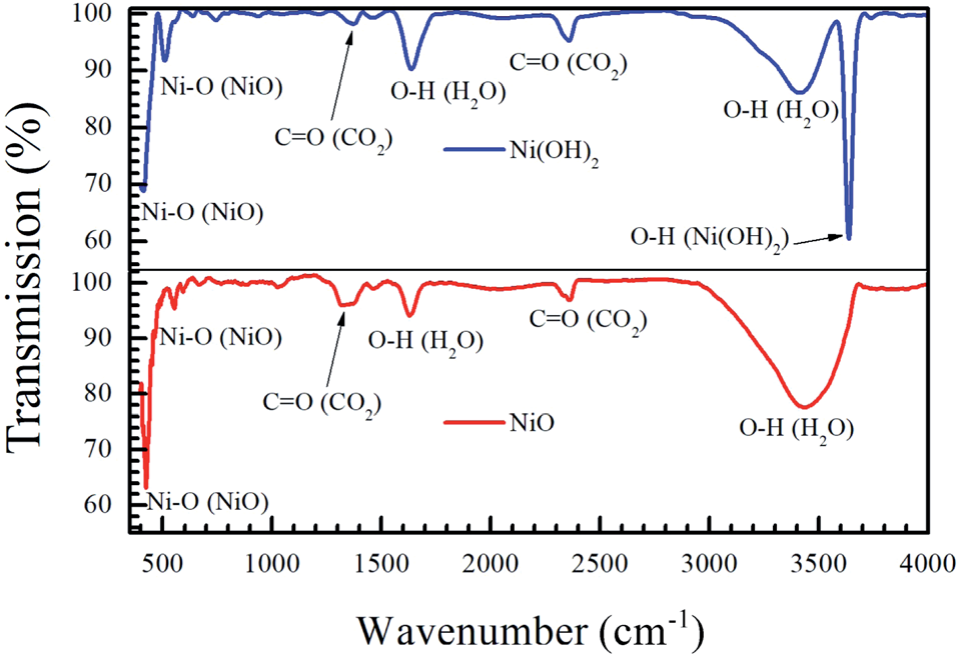
Fourier-transform infrared response of the precursor Ni(OH)_2_ and annealed NiO nanoparticles shows that the oxidation from hydroxide to oxide is complete by annealing at 400 °C for 2 h.

Fig. 3 shows the infrared absorption behaviour of the precursor Ni(OH)_2_ and the NiO formed after annealing. The intense peak at 3634 cm^−1^ corresponds to the hydroxide stretching mode in Ni(OH)_2_.^42^ The sharp peaks present in both samples at 470 and 520 cm^−1^ correspond to the Ni–O stretching mode in NiO and Ni(OH)_2_.^43^ The broad peak centered at 3440 cm^−1^ and the peak at 1635 cm^−1^ correspond to the stretching and bending mode of the O–H bond in H_2_O, while the peak at 1390 and 2400 cm^−1^ correspond to the asymmetric and symmetric stretching mode of the O=C bond in CO_2_.^42^ Ultrafine NiO and Ni(OH)_2_ nanoparticles are known to adsorb atmospheric CO_2_ and water vapour, leading to the detection of these compounds in both materials.^42–44^ The absence of the peak at 3634 cm^−1^ in the NiO sample indicates that the oxidation of the nickel hydroxide to nickel oxide is complete, and that there is no unreacted hydroxide left.

**Fig. 4:**
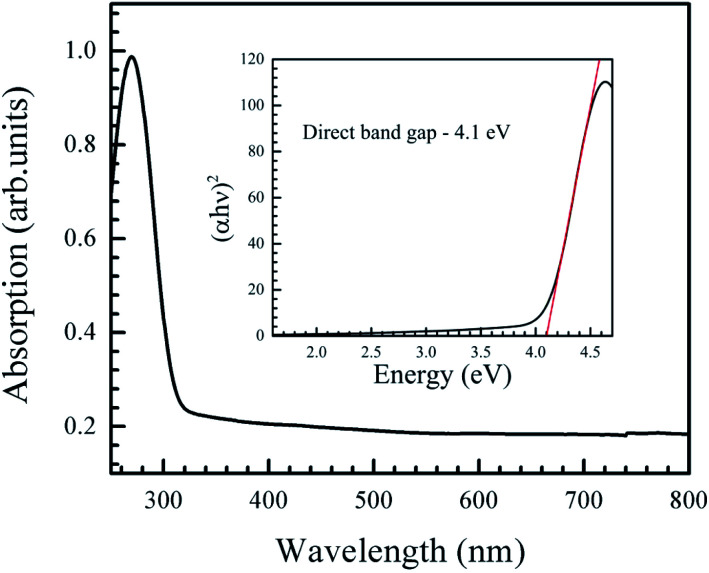
Optical absorption spectrum of the NiO nanoparticles. Inset is the Tauc plot, showing a direct band gap of 4.1 eV.

The absorption spectrum of the nanoparticles (Fig. 4) displayed a sharp edge at around 290 nm. The optical gap was obtained from the Tauc plot, where (*αhν*)^2^ was plotted against *hν*, where *h* is the Planck's constant, *α* is the absorption coefficient, *ν* is the frequency of the incident light. The Tauc plot (inset in Fig. 4) showed a direct gap value of 4.1 eV, which is in agreement with other reports.^10,45^

**Fig. 5:**
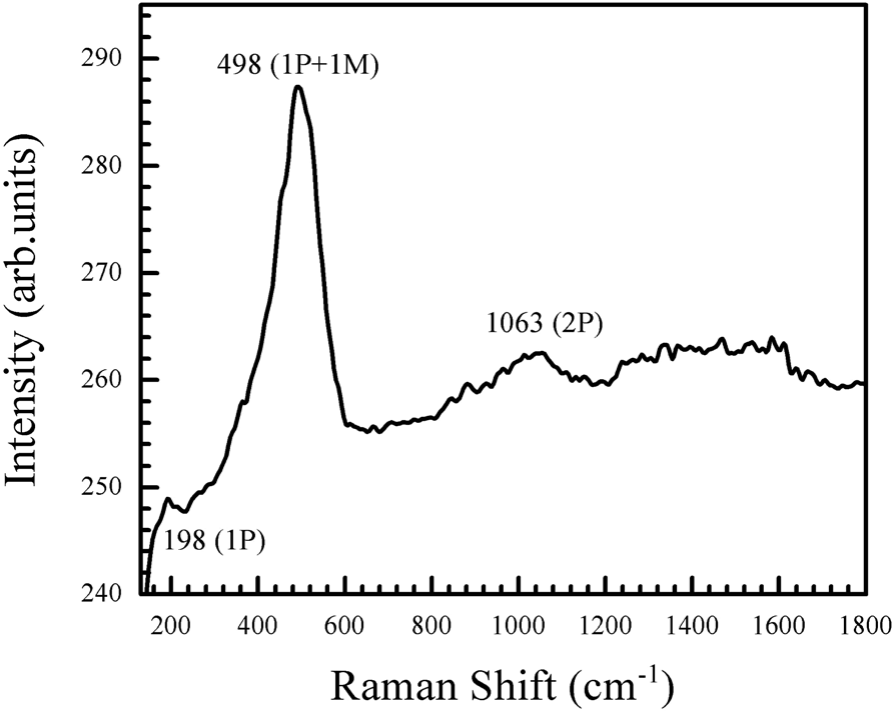
Raman spectrum of the NiO nanoparticles. There is a strong defect peak at 498 cm^−1^, corresponding to Ni vacancies in the particles.

Stoichiometric NiO is green in colour, while the annealed powder obtained in this work is black in colour. To understand the nature of defects present in the NiO nanoparticles, Raman spectrum was recorded and is shown in Fig. 5. The intense peak at 498 cm^−1^ is a defect peak corresponding to Ni vacancies (V_Ni_).^46,47^ This agrees with previous studies which indicate that defect rich NiO has a strong first order scattering peak between 400 and 600 cm^−1^, and this scattering mode is absent in stoichiometrically pure NiO.^13^ A forbidden phonon mode is observed at 198 cm^−1^. This mode is reported to be absent in NiO single crystals, and it arises due to the lowered symmetry of the lattice that occurs as a consequence of defects caused by oxygen composition. A two-phonon mode is also observed at 1063 cm^−1^.^46^

### 3.2 Ink characterisation

To formulate a stable and printable nanofluid using the NiO nanoparticles, a variety of polar and non-polar solvents were tested. The nanoparticles were dispersed through bath ultrasonication. Hexane and acetone produced dispersions that settled at the bottom of the vessel within 5 s of stopping the ultrasonication, while ethanol and water produced dispersions that were slightly more stable – lasting for nearly 15 s. EG, however, produced dispersions that did not settle for several weeks. EG has been previously used to make printable inks, owing to its high viscosity and boiling point.^48–50^ Furthermore, upon settling, the nanoparticles can be re-dispersed in the medium and reused, without loss of functionality. Hence, EG was chosen as the medium to produce the NiO dispersion. No surfactants were used in the formulation of the NiO ink. This is because surfactants tend to affect the electrical conductivity of the printed pattern and need to be removed post printing, either by annealing or by the use of specific chemical treatments. NiO-based inks have been fabricated through bath and probe ultrasonication using mixtures of solvents, such as propylene glycol, isopropanol and propanol, but these tend to have millimetre sized inhomogeneities while printing or are not printable.^51^

The rheological properties of the nanofluid are important for its use as a printable ink. The surface tension of the formulated ink was found to be 45.9 ± 0.2 mN m^−1^. This lies within the range of optimal surface tension of inks used in DOD systems, which is 30–60 mN m^−1^.^7^ NiO-inks with surface tension values outside this permissible limit tend to spread outside the bounds of the desired pattern.^37^

**Fig. 6:**
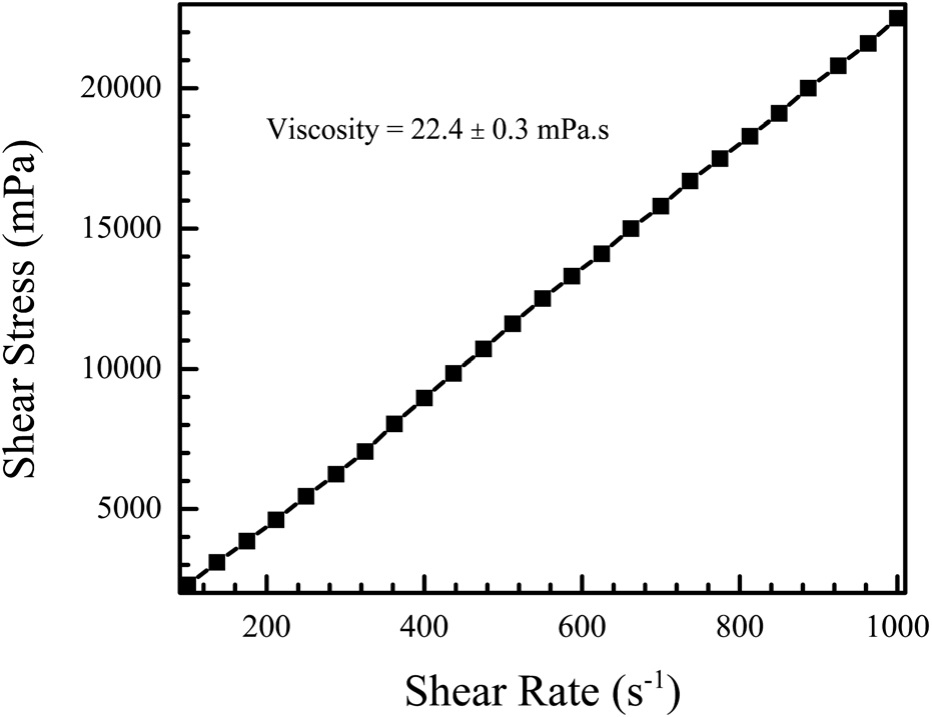
Shear stress *vs.* shear rate measurement of the NiO nanofluid. The fluid exhibits Newtonian behaviour and the viscosity, calculated from the slope of the plot, was found to be 22.4 ± 0.3 mPa s.

Fig. 6 shows the shear stress *versus* shear rate plot for the NiO nanofluid. The plot is linear, characteristic of Newtonian fluids, and the slope gives the viscosity, which was found to be 22.4 ± 0.3 mPa s. This is slightly higher than the normal range for use with DOD systems, *i.e.* between 1 and 20 mPa s.^52^

The viscosity and surface tension values can be used to calculate a dimensionless number called the Ohnesorge number (Oh), which represents the ease of droplet formation from a nozzle during inkjet printing.^9^ It is defined as1
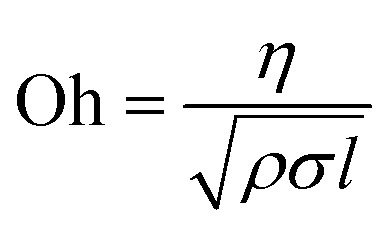
where *η* is the dynamic viscosity of the ink (22.4 mPa s), *ρ* represents its density (1.3 g cm^−3^), *σ* represents the surface tension (45.9 mN m^−1^) and *l* is the nozzle diameter of the printer. The inverse of the Ohnesorge number is called the *Z* number (*Z*). *Z* must lie between 1 and 10 to be printable.^53^ For low *Z* values, the highly viscous nature of the fluid does not allow drop ejection from the nozzle, and for high *Z* values, excessive satellite droplet formation is observed. Commercial inkjet printers have nozzle diameters ranging from 10 μm to 150 μm.^54^ For a printer with a nozzle diameter of 10 μm, the ink we formulated has a *Z* value of 1.09, and for a nozzle diameter of 150 μm, we get a *Z* value of 4.22. Both are well within the printable range. Therefore, the NiO ink produced here is jettable through standard commercial inkjet printers.

### 3.3 Characterisation of the dropcast and printed patterns

Contact angle is an important parameter in determining the fidelity of the printed patterns. If the ink is hydrophobic, it would not wet the substrate. However, too low a contact angle would mean that the ink would spread and pattern fidelity cannot be established. The contact angle of the NiO ink on glass was 47.3 ± 0.3°, and on the AZO coated glass substrate was 69.0 ± 0.1°. Thus, both substrates are suitable for printing. Fig. S3† shows representative contact angle images on a cleaned glass slide and AZO coated glass. Table T1† lists the individual contact angle measurements and the mean and standard deviation. Ten measurements were taken for each substrate and averaged.

The self-assembly of the nanoparticles during drying is also important. During drying, the particles tend to agglomerate at the circumference of the ink drop. This phenomenon is known as the coffee ring effect.^55^ This typically results from fluid flow from the center to the drop edge, which is pinned to the substrate, during solvent evaporation. The coffee ring effect can potentially affect the connectivity of the printed nanoparticles, as it causes concentration gradients within the pattern. This effect can be controlled by manipulating the surface tension of the ink or through the bed temperature used in printing.^8^ The profiles of the dropcast patterns were observed as a function of the substrate temperature during printing.^9^ The coffee ring effect was observed in all samples, and its extent appeared largely invariant with the substrate temperature, as observed in S4 of the ESI.† However, it was observed that lower substrate temperatures favoured “spreading” of the pattern (the pattern occupied a larger surface area and exhibited large variances in particle concentrations) while higher substrate temperatures showed patterns with high particle concentrations in small areas, indicating that the latter is more suitable for achieving optimal connectivity (and hence conductivity) within a desired area.†

**Fig. 7:**
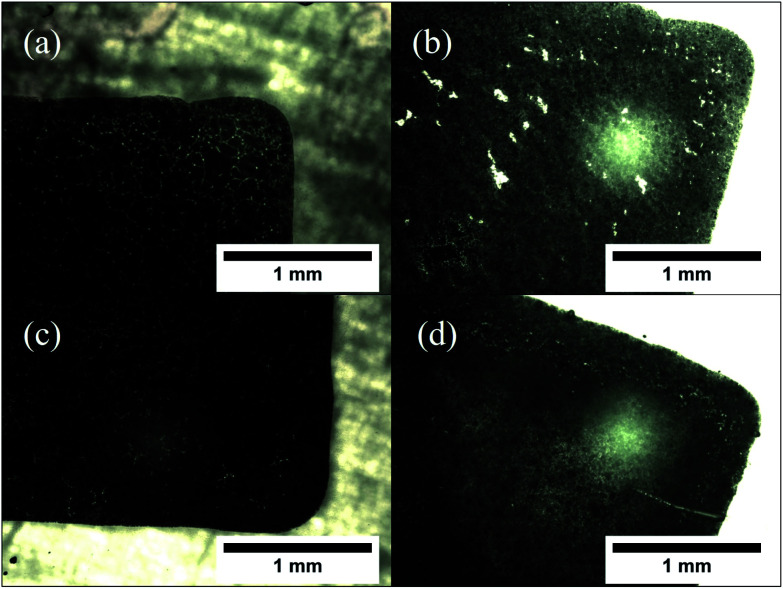
Optical micrographs, taken at a magnification of 50×, show segments of the printed square patterns on glass. (a) Printed at 30 °C, before annealing. (b) Printed at 30 °C, after annealing. (c) Printed at 60 °C, before annealing. (d) Printed at 60 °C, after annealing. Less cracks are visible in the pattern printed at higher temperature.

The morphology of the patterns printed on glass slides appeared to be invariant with the temperature of printing. However, upon annealing, it was found that patterns printed at lower temperatures (30 and 40 °C) exhibited a proclivity to form cracks in the printed film. This was not observed with the patterns printed at higher temperatures (50 and 60 °C). This is visualized in the optical micrographs in Fig. 7. It is expected that the spreading of the pattern at lower substrate temperatures causes specific regions of low particle concentration, which get accentuated by the sintering process into visible cracks. Based on this, the optimal bed temperature for deposition was fixed at 60 °C.

The ink did not wet the AZO coated glass as effectively as plain glass, as evidenced by comparing the contact angles. To ensure connectivity of the NiO nanoparticles while printing on AZO coated glass, the printing was done in two passes instead of one. Table 2 shows the thickness and surface roughness of the printed patterns on the glass and AZO coated substrates after printing and subsequent annealing. The thickness of the printed patterns on glass and on AZO coated glass was found to be largely invariant with the temperature. The surface roughness reduced with increasing printing substrate temperature, as observed with the samples printed on glass. The sample printed on AZO coated glass and annealed at 250 °C was observed to have the most uniform surface.

**Table 2:** Surface roughness and thickness of the printed films

Substrate	Printing *T* (°C)	Number of passes	Annealing *T* (°C)	Thickness (μm)	Surface roughness (μm)
Glass	30	1	250	1.12 ± 0.66	0.66 ± 0.36
Glass	60	1	250	1.01 ± 0.28	0.18 ± 0.04
AZO Glass	60	2	200	1.10 ± 0.13	1.01 ± 0.18
AZO Glass	60	2	250	1.15 ± 0.31	0.66 ± 0.04
AZO Glass	60	2	300	1.14 ± 0.32	1.31 ± 0.19

### 3.4 Electrical characterisation of the heterojunction

**Fig. 8:**
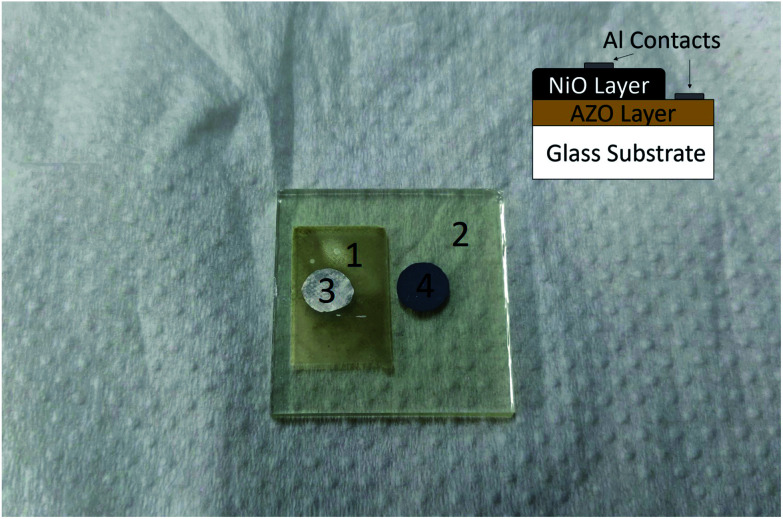
Picture of the device. The components are: (1) printed p-type NiO layer (1.5 × 1 cm) (2) n-type AZO coated glass (2.5 × 2.5 cm) (3) and (4) aluminum contacts (6 mm diameter and spaced 12 mm apart). Inset graphic shows a cross-sectional representation of the device structure.

**Fig. 9:**
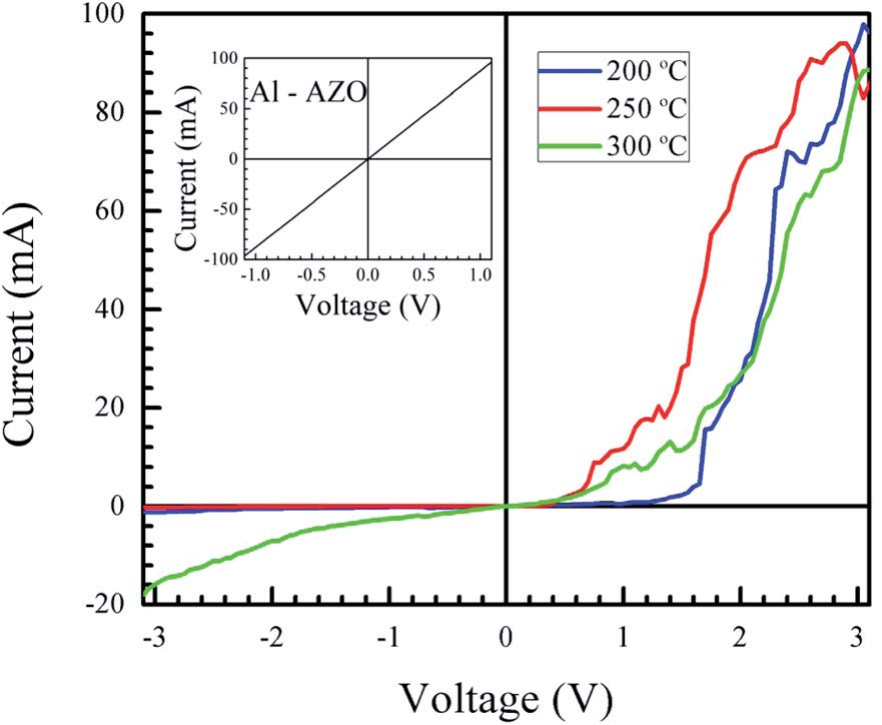
Current–voltage characteristics of the devices annealed at different temperatures. All three samples exhibit rectification arising from the junction between NiO and AZO. The inset figure shows the current–voltage characteristics of the AZO–Al junction, which exhibits ohmic behaviour.

Fig. 8 shows the fabricated heterojunction after printing NiO, drying, annealing, and deposition of aluminum contacts. The dimensions were kept constant across different devices. Fig. 9 shows the current–voltage characteristics of the devices annealed at 200, 250, and 300 °C. The aluminum–AZO junction was found to be ohmic, as seen in the inset in Fig. 9. The NiO–aluminum junction is also known to exhibit ohmic behaviour.^56^

**Fig. 10:**
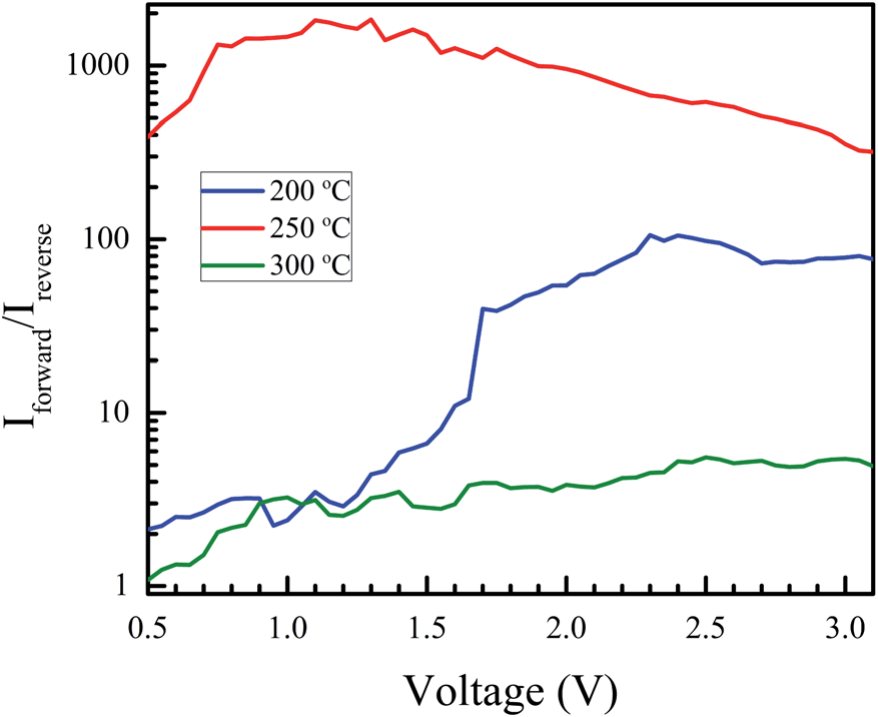
Extent of rectification *vs.* voltage for devices annealed at 200, 250 and 300 °C, plotted as a ratio of the forward to reverse bias currents, as a function of applied voltage. The rectification is maximum for the sample annealed at 250 °C.

Rectification is observed for all three annealing temperatures. Its extent was studied by plotting the ratio of the magnitude of the forward bias current to the reverse bias current as a function of the bias voltage. This is shown in Fig. 10. The best rectification was obtained at an annealing temperature of 250 °C, and the lowest was observed at 300 °C. The rectification ratio of the sample annealed at 250 °C (in the thousands) is one or two orders of magnitude higher than those previously reported for solution-processed NiO–ZnO systems.^57–59^ The device annealed at 300 °C showed an increased leakage current and the lowest extent of rectification. This can be attributed to shorting of the circuit due to crack formation at higher annealing conditions. To test this, we measured the resistance of the bare AZO substrate, as a function of temperature. The data is shown in Fig. S5 of the ESI.† The increase in resistance of the AZO films occurs due to dewetting and crack formation in the film due to the annealing. Cracks also form in the printed NiO films leading to short-circuiting of the film when Al is deposited over it, causing hindered performance and increased leakage current.

To further understand the electrical behaviour, the *I*–*V* plots were curve fitted with the equation for current in a pn junction with an internal resistive component2
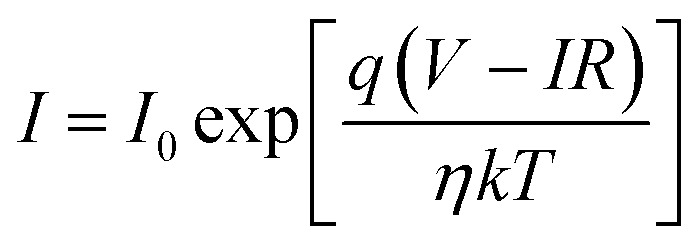
where *I* is the forward bias current, *I*_0_ is the reverse saturation current, *V* is the applied voltage, *R* is the series resistance of the junction, *η* is the diode ideality factor and *T* is the temperature (300 K).^60^ The junction barrier height was obtained by modeling *I*_0_ using the following equation^61^3
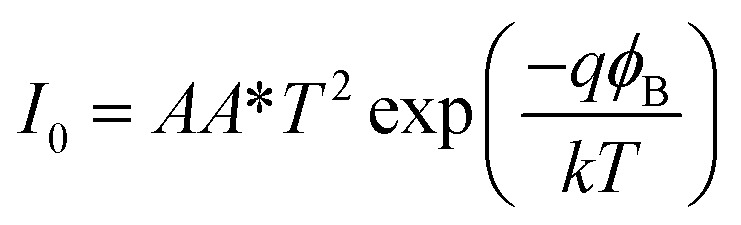
Here, *A* is the diode contact area, A* is the Richardson constant and *ϕ*_B_ is the barrier height of the junction. The values of the curve fit parameters and *ϕ*_B_ are shown in Table 3.

**Table 3:** Parameters obtained from fitting the *I*–*V* curves using eqn (2) and (3)

Annealing *T* (°C)	*I*_0_ (mA)	*R* (Ω)	*η*	*ϕ*_B_ (eV) (curve fit)	*ϕ*_B_ (eV) (Norde's method)
200	0.05 ± 0.02	8.9 ± 0.4	11.4 ± 0.4	0.65	0.57
250	2.59 ± 0.23	12.8 ± 0.2	16.9 ± 0.3	0.55	0.52
300	0.62 ± 0.19	9.6 ± 1.1	17.4 ± 1.7	0.59	0.57

Similar ideality factors have been reported for solution processed NiO–ZnO systems.^57^ NiO–ZnO systems fabricated through radio-frequency magnetron sputtering have been shown to have slightly higher *η* values.^62^ Ideality factors greater than 2 in pn heterojunctions have previously been attributed to non-linearity in the current–voltage behaviour of the metal–semiconductor junctions.^63^ However, both the Al–AZO and the Al–NiO junctions have been shown to be ohmic. Therefore, this cannot be the cause for the large ideality factors in our system. Trap assisted recombination and interfacial defects have also been known to cause high ideality factors.^58,64^ The NiO nanoparticles were shown to have a high defect density, as observed through its Raman spectrum (Fig. 5). Therefore, defect mediated conduction in the NiO layer and the presence of interface states are likely the origin of the observed ideality factors.

As the current–voltage behaviour exhibited rectification with high ideality factors (*η* > 2), a modified version of Norde's method was used to analyze the data.^65^ The method is shown through the following equations4
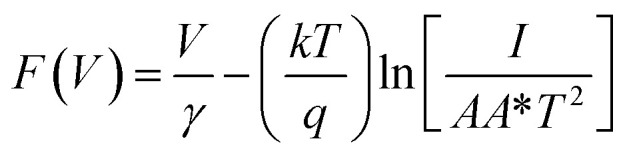
5
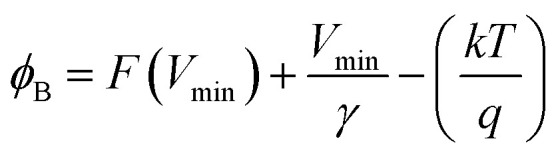
6
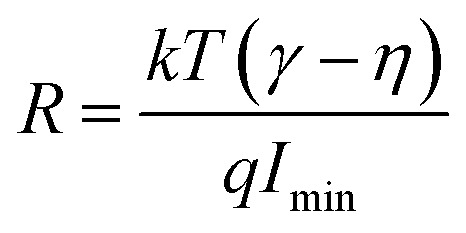
Here, *F*(*V*) is Norde's function, *γ* is an arbitrarily chosen parameter greater than the ideality factor, *V*_min_ and *I*_min_ are the values of *V* and *I* at the minimum of *F*(*V*).

**Fig. 11:**
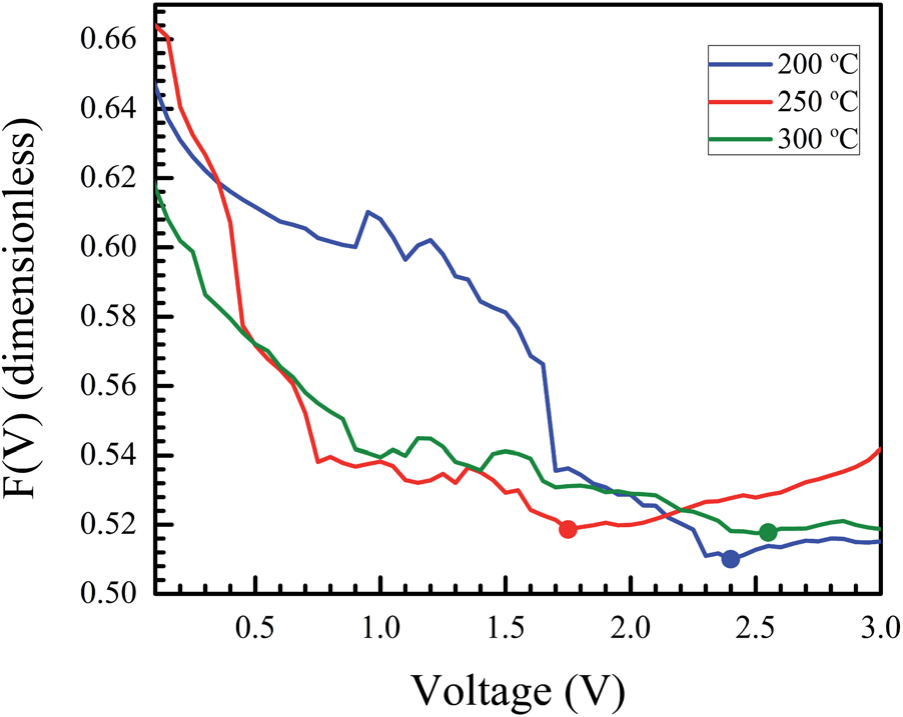
Norde's function plotted for samples annealed at different temperatures. The minimum in the plot (marked by a dot for each curve) is used to calculate the barrier height for the junction.

After identifying the minimum of *F*(*V*), depicted in the plot in Fig. 11, eqn (5) and (6) were used to calculate the junction barrier heights. The values of *ϕ*_B_ are shown in Table 3 and are in good agreement with the values obtained using eqn (3). These values of *ϕ*_B_ are very similar to the difference in work functions of NiO (5.2 eV) and AZO (4.65 eV).^41^ The *ϕ*_B_ value of the junction annealed at 250 °C was in closest agreement with the theoretically predicted value (0.55 eV). The values are also in good agreement with reported values of junction barrier heights for solution processed NiO–ZnO systems.^58^

## 4 Conclusions

Phase pure NiO was synthesised through a bottom-up approach, as confirmed by X-ray diffraction and Fourier-transform infrared spectroscopy. The Raman spectrum of the material revealed a high defect density. The absorption spectrum was studied and the optical gap obtained was in line with previous reports. A stable ink was prepared using bath ultrasonication, with EG as the dispersing medium. The viscosity and surface tension were measured and the calculated Ohnesorge number was within the optimal range for inkjet printing. Dropcast patterns of the ink showed that the ink spread while bring dropcast at lower temperatures, leading to significant inhomogeneities in particle distribution which caused microscopic cracks to form in the film. The contact angle of the ink on glass and AZO-coated glass was found to be optimum for printing. The ink was printed on the AZO-coated glass, and it was observed that the surface roughness was minimal for the devices annealed at 250 °C. A heterojunction was fabricated and aluminum contacts were deposited on the NiO and the AZO layers. The current–voltage response was studied, showing rectifying behaviour, and the annealing temperature for maximum rectification was identified. The ideality factor, series resistance, and barrier height of the junction were calculated through curve-fitting. The junction barrier height was also obtained using Norde's method, and the values were in close agreement with those obtained through curve-fitting and with the theoretically predicted value for NiO–AZO heterojunctions. It was observed that annealing the heterojunction at 250 °C formed a junction with the highest extent of rectification. Future work will focus on using these printed heterojunctions for sensing applications.

## Conflicts of interest

The authors declare no competing financial interests.

## Supplementary Material

RA-010-C9RA08466E-s001
