# Supplementary material for: Metal oxide heterojunctions using a printable nickel oxide ink
Source: RSC Adv. 2020 Jan 23;10(7):3951–9. doi: 10.1039/c9ra08466e (PMC9048838; doi:10.1039/c9ra08466e)
Supplement: RA-010-C9RA08466E-s001 [file RA-010-C9RA08466E-s001.pdf]

Electronic Supplementary Material (ESI) for RSC Advances.
This journal is © The Royal Society of Chemistry 2020

Metal Oxide Heterojunctions using a p-type Nickel Oxide Ink

Hari Ramachandran,[a] Mohammad Mahaboob Jahanara,[a] Nitheesh M. Nair,[a&b] and P. Swaminathan[*a]*

[a] Electronic Materials and Thin Films Laboratory, Department of Metallurgical and Materials Engineering, [b] Organic Electronics Group, Department of Electrical Engineering, Indian Institute of Technology Madras, Chennai 600036

[*] Corresponding Author - swamnthn@iitm.ac.in

Electronic Supplementary Information

Description of the printer and the printing process

Figure S1 shows the custom-built direct writer setup used in this work. A DC stepper motor controls the extrusion of the ink from the syringe. The speed of the syringe can be controlled, which in turn controls the volumetric extrusion rate. Horizontal stepper motors control the motion of the substrate bed and syringe (in mutually perpendicular directions). The desired pattern is loaded as a stereolithographic file or a G-code program.

Prior to printing, the ink is loaded into the syringe and care is taken to ensure that the air bubbles are eliminated. The substrate was fixed to the bed and the requisite pattern is loaded into the software. The pattern is then 'written' by the needle tip, which extruded ink at a pre-defined rate. In cases where printing is to be done at higher temperatures, a dwell time of 10 minutes is allowed both before and after the printing process to equilibrate the substrate and bed temperature and to facilitate evaporation of the solvent.

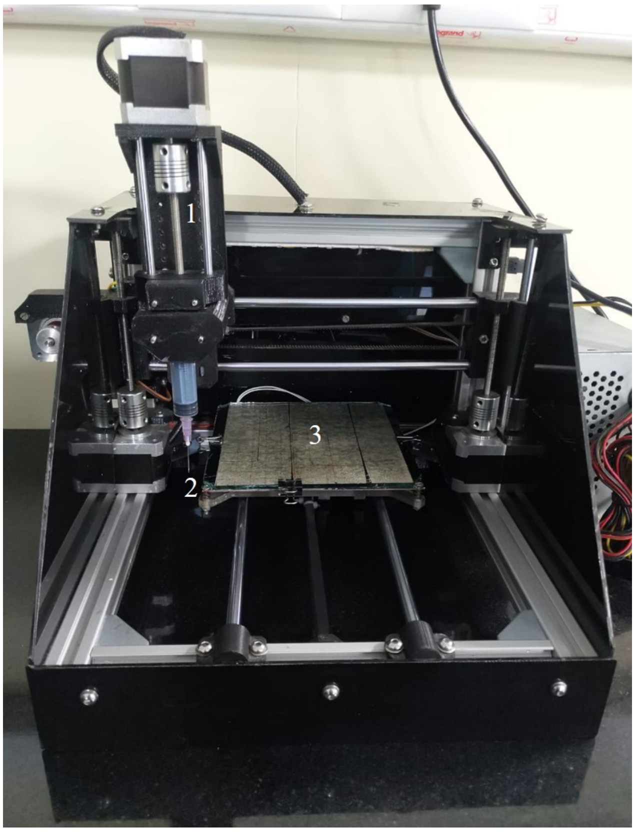

S1 The custom-built direct writer used for printing the nanofluid. The components are - 1. DC stepper motor, 2. Syringe and needle and 3. Substrate bed.

SEM images of NiO nanoparticles

Figure S2 shows low magnification SEM images (at two different magnifications) of the NiO nanoparticles. Agglomeration is observed, which is characteristic of nanoparticles due to their surface area. Surfactants can be used to mitigate agglomeration, but these are often challenging to eliminate and can interfere with the electronic properties and functionality of the layer if removal is incomplete.

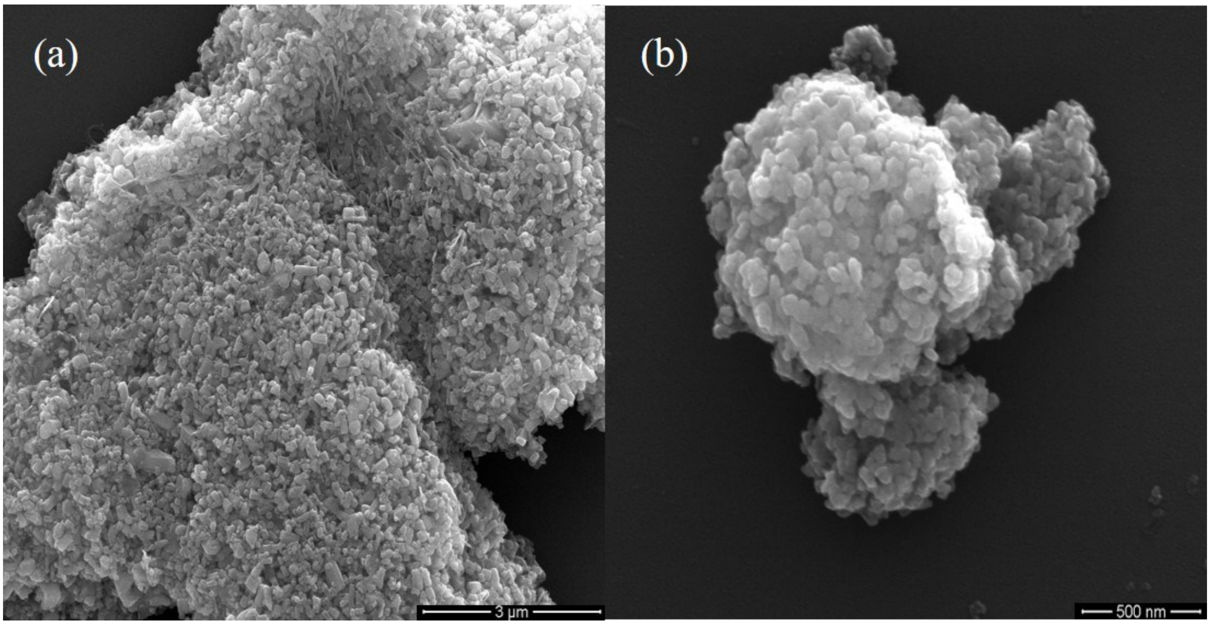

S2 SEM micrographs of the NiO at two different magnifications. (a) and (b) show agglomeration of the individual nanoparticles, in the absence of surfactants.

Contact angles of nanofluid on substrates

Figure S3 shows a representative figure of the contact angle of the NiO nanofluid on a cleaned glass substrate and the AZO glass substrate. The table T1 shows the values of the contact angles on the two substrates. across several readings. The mean contact angle was found to be 47.3 ± 0.3° for the NiO ink on glass and 69.0 ± 0.1° for the NiO ink on AZO glass.

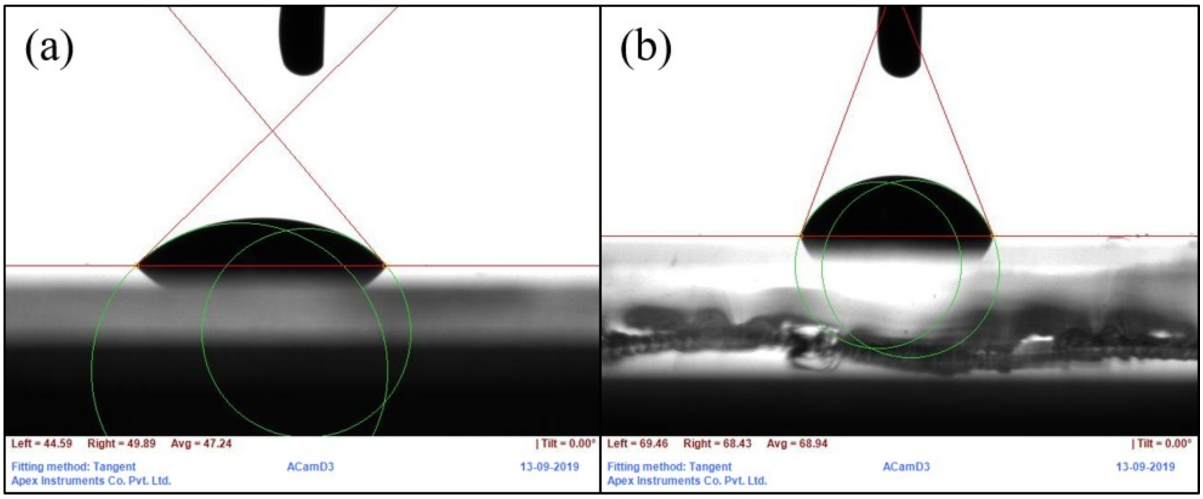

S3 Representative images of contact angle measurement of the NiO nanofluid on (a) soda-lime glass and (b) AZO coated glass, showing its hydrophilic nature. Ten measurements were taken for each sample and then averaged. The data is shown in Table T1.

T1 Contact angle measurements of NiO nanofluid on glass. Ten measurements were made and averaged.

**S.no.**	**Contact angle – glass (°)**	**Contact angle – AZO glass (°)**
1	47.2	68.9
2	47.2	68.9
3	47.8	69.1
4	47.2	69.1
5	47.2	69.1
6	46.7	68.9
7	47.2	69.1
8	47.8	68.9
9	47.2	69.1
10	47.2	69.1
**Mean ± Std. Dev.**	47.3 ± 0.3	69.0 ± 0.1

Optical profilometry of dropcast patterns

Figures S4 show optical profilometry studies of 2 □l of the NiO ink dropcast at 30 and 60 °C respectively and dried in a hot air oven at 120 °C for 1 hour. There does not appear to be a major difference in the extent of the coffee ring effect, as both samples have significant accumulation of NiO at their respective peripheries. However, it can be seen that the sample dropcast at 30 °C was found to occupy a larger area despite the dropcast volume and concentration being identical - the dried pattern had a diameter of close to 3.5 mm while the pattern dropcast at 60 °C had a diameter of almost 2.5 mm. This indicates that printing at higher temperatures is more likely to provide patterns that conform to a desired shape without spreading.

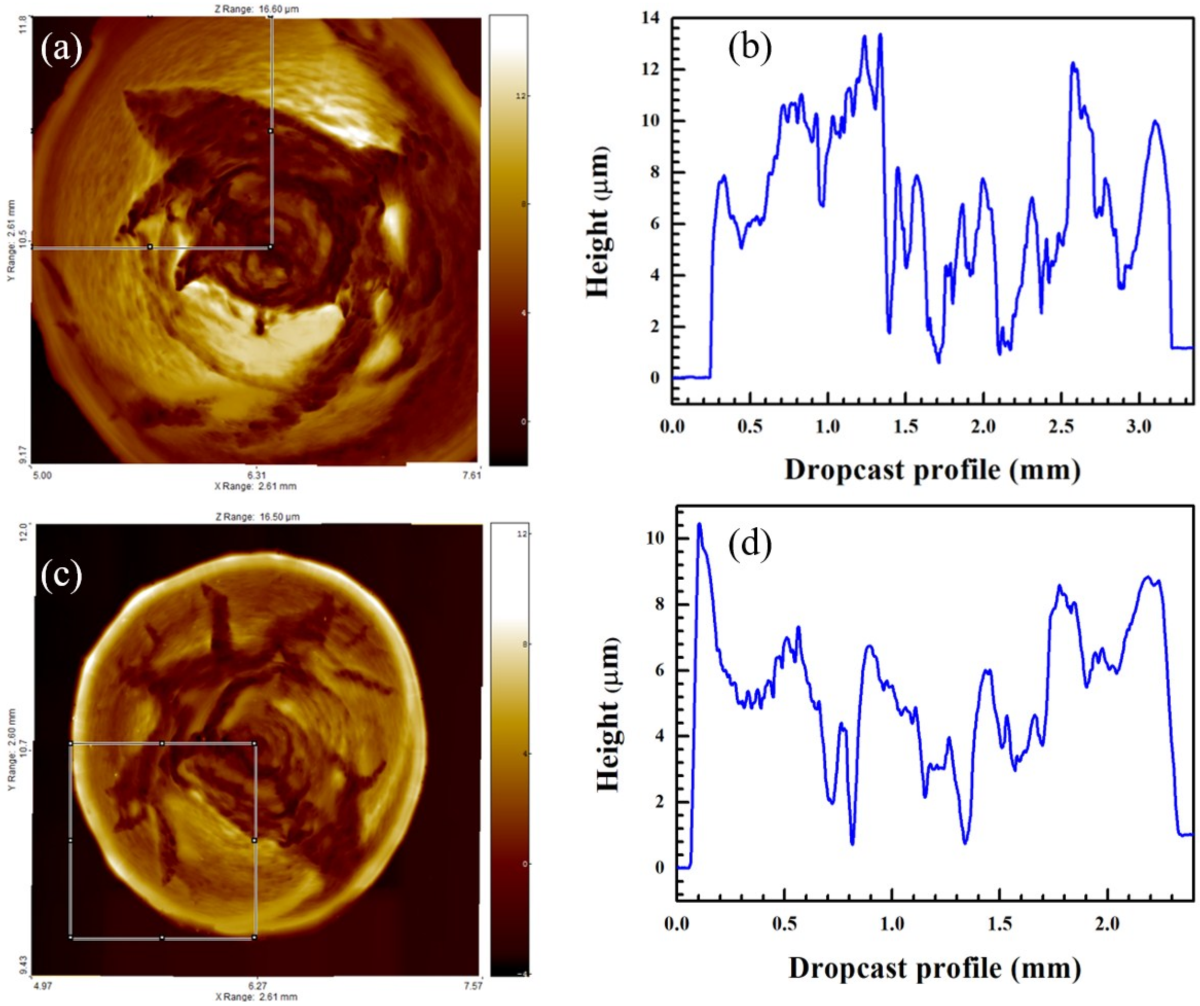

S4 – Optical profile data of the dropcast NiO ink. (a) Image of ink dropcast at 30 °C, dried in hot air oven at 120 °C for 1 hour. (b) Line profile of the dried drop across a diameter. (c) Image of ink dropcast at 60 °C, dried in hot air oven at 120 °C for 1 hour. (d) Line profile of the dried drop across a diameter.

Temperature dependent resistance variation of AZO coated glass

Figure S5 shows the variance of the sheet resistance of the bare AZO coated glass slides with the annealing temperature. The resistance increases monotonically from 200 to 300 °C, and increases significantly at 350 °C. This variation is responsible for the increase in leakage current that is observed in devices that were annealed at 300 °C compared to devices that were annealed at lower temperatures.

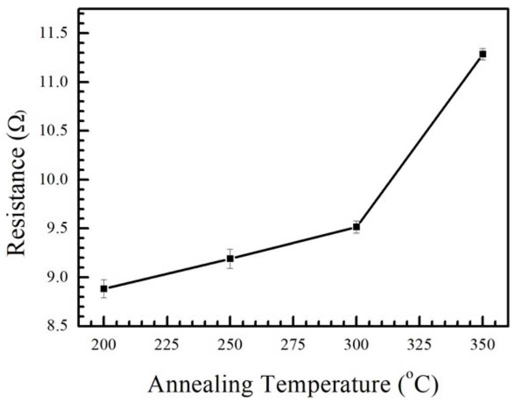

S5 Variation of the resistance of the AZO glass with annealing temperature. Beyond 300 ℃, the resistance increases drastically, and this is responsible for the increased leakage currents seen at the higher temperature.
